# Author Correction: A functionally conserved *STORR* gene fusion in *Papaver* species that diverged 16.8 million years ago

**DOI:** 10.1038/s41467-022-31568-x

**Published:** 2022-06-29

**Authors:** Theresa Catania, Yi Li, Thilo Winzer, David Harvey, Fergus Meade, Anna Caridi, Andrew Leech, Tony R. Larson, Zemin Ning, Jiyang Chang, Yves Van de Peer, Ian A. Graham

**Affiliations:** 1 Centre for Novel Agricultural Products, Department of Biology, University of York, York, YO10 5DD UK; 2 Bioscience Technology Facility, Department of Biology, University of York, York, YO10 5DD UK; 3 The Wellcome Sanger Institute, Wellcome Genome Campus, Hinxton, Cambridge CB10 1SA UK; 4 Department of Plant Biotechnology and Bioinformatics, Ghent University, and VIB Centre for Plant Systems Biology, 9052 Ghent, Belgium; 5 Centre for Microbial Ecology and Genomics, Department of Biochemistry, Genetics and Microbiology, University of Pretoria, Pretoria, 0028 South Africa; 6 College of Horticulture, Academy for Advanced Interdisciplinary Studies, Nanjing Agricultural University, Nanjing, China

**Keywords:** Genome evolution, Secondary metabolism, Plant evolution

Correction to: *Nature Communications* 10.1038/s41467-022-30856-w, published online 07 June 2022.

In the original version of this Article, the positions of “Morphine” and “Codeine” and the associated chemical structures in the morphinan pathway in Fig. 1a were swapped. These have been corrected in both the PDF and HTML versions of the Article.

