# Supplementary material for: A functionally conserved STORR gene fusion in Papaver species that diverged 16.8 million years ago
Source: Nat Commun. 2022 Jun 7;13:3150. doi: 10.1038/s41467-022-30856-w (PMC9174169; doi:10.1038/s41467-022-30856-w)
Supplement: Supplementary file 1 — Supplementary Information [file 41467_2022_30856_MOESM1_ESM.pdf]

A functionally conserved *STORR* gene fusion in *Papaver* species that diverged 16.8 million years ago

Catania *et al*.

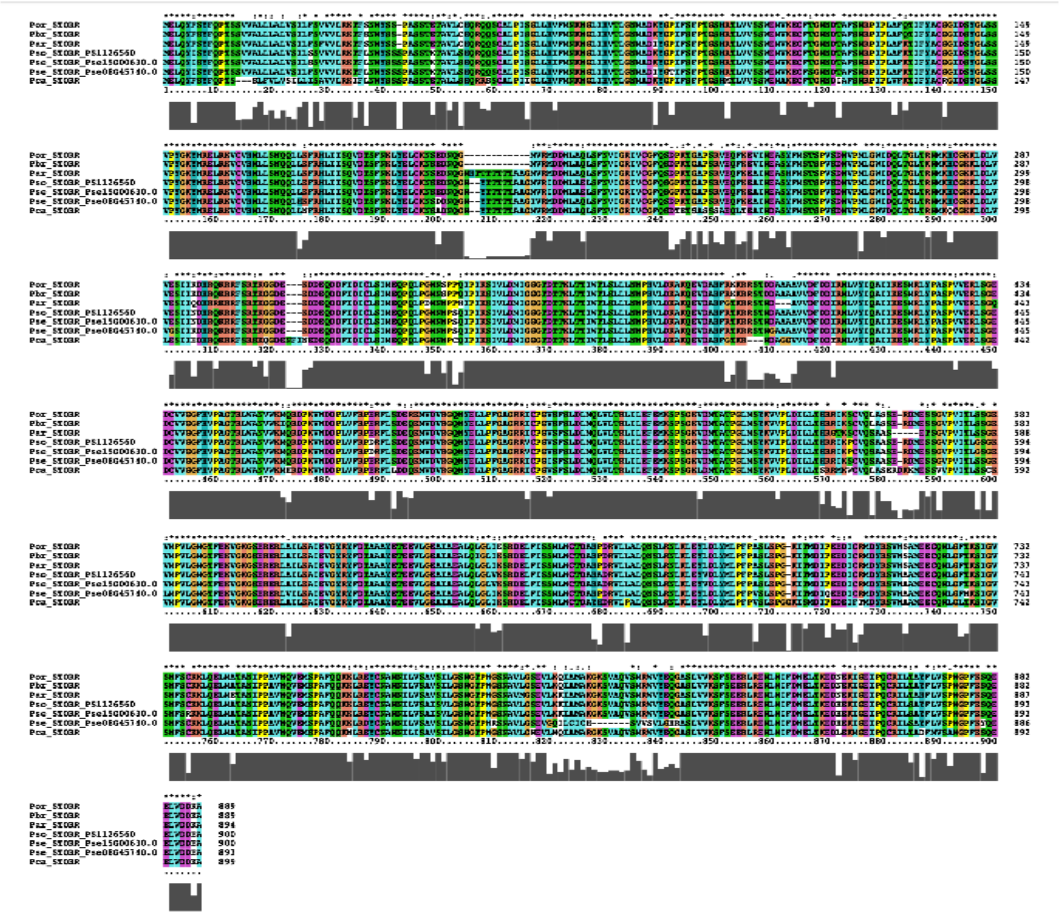

Supplementary Figure 1. Multiple sequence alignment of STORR proteins from six Clade 2 *Papaver* species. Source data are provided as a Source Data file.

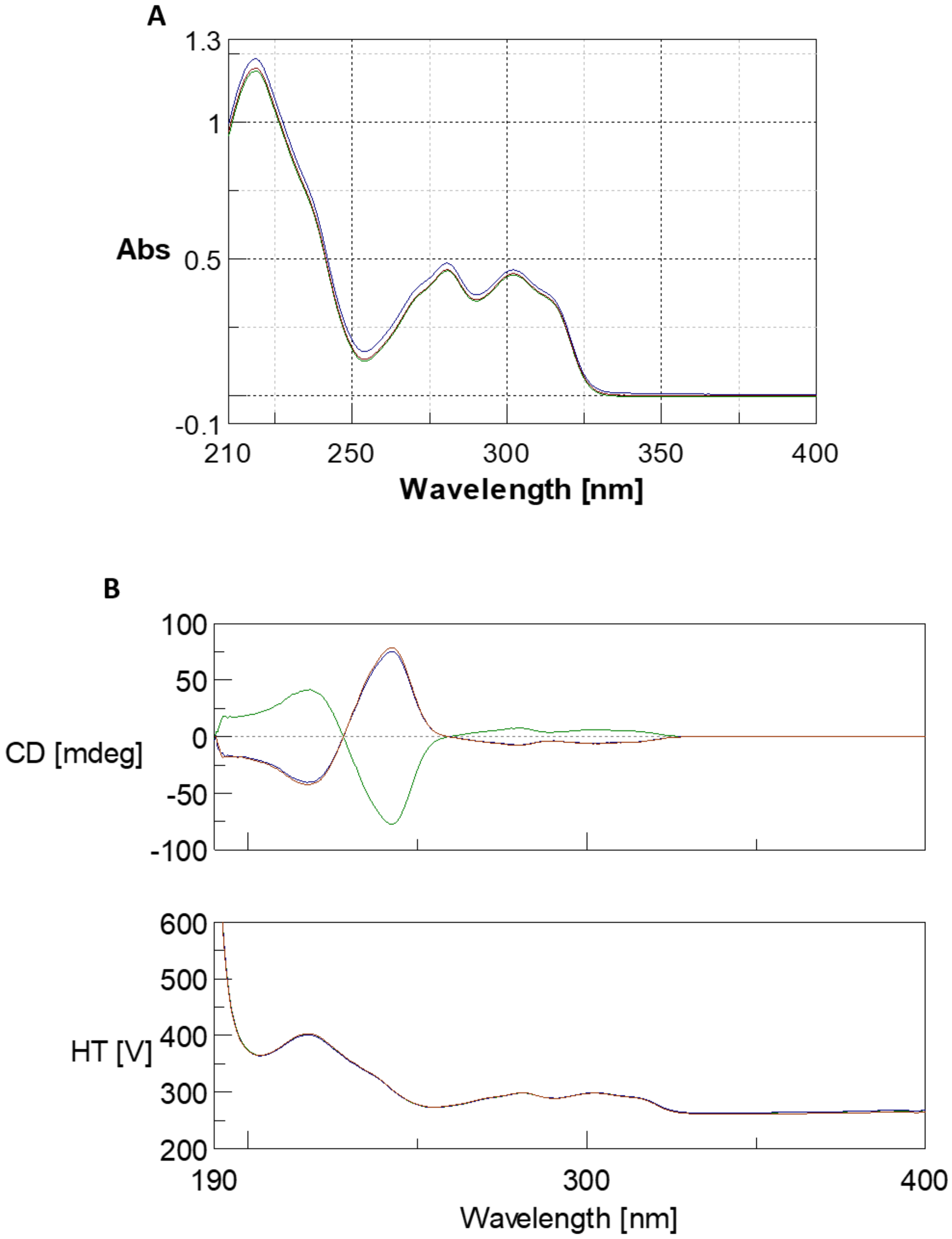

Supplementary Figure 2. UV spectra and CD spectra for *G. flavum* and *P. californicum* extracts

A. UV spectra for Authentic (*S*)-glaucine standard (blue), *Glaucium flavum* glaucine preparative HPLC extract (green) and Papaver californicum preparative HPLC extract (brown). **B.** Overlay of the Circular dichroism (CD) spectra for Authentic (*S*)-glaucine standard (brown), *Glaucium flavum* glaucine preparative HPLC extract (blue) and Papaver californicum preparative HPLC extract (green). The concentration of samples run 0.125mg/ml. Source data are provided as a Source Data file.

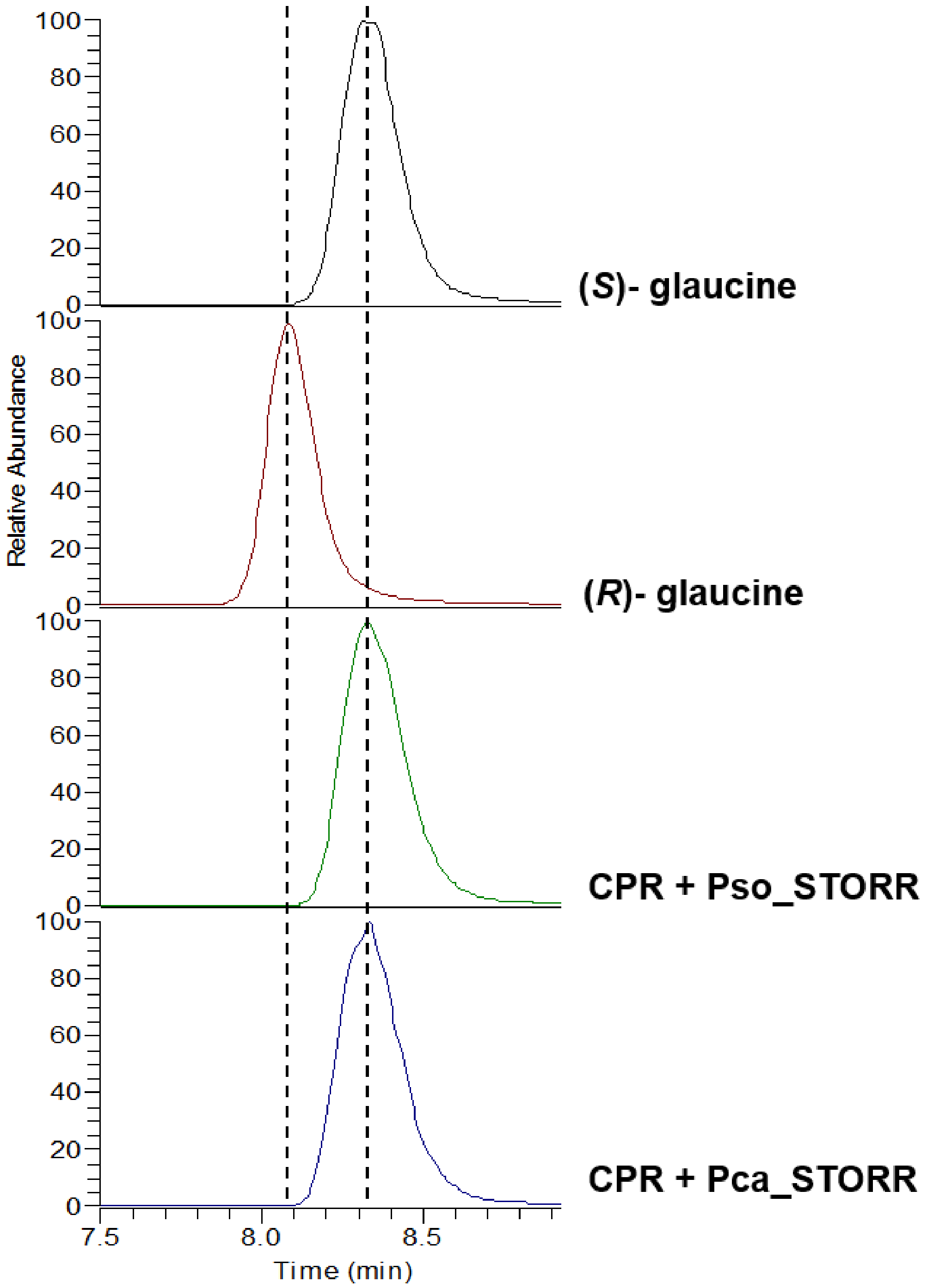

Supplementary Figure 3. Enzyme assay with (*S*)-glaucine substrate

Substrate specificity of the microsomal extracts from *S. cerevisiae* expressing the STORR protein from *P. californicum* alongside *P. somniferum* (control) were assayed with (*S*)-glaucine as a substrate. Relative abundance is used to show the glaucine epimers present in the sample. Glaucine standards- (*S*)-glaucine (black) (*R*)-glaucine (red), pESC-TRP+ CPR+ Pso STORR (green) and pESC-TRP+ CPR+ Pca STORR (blue). Source data are provided as a Source Data file.

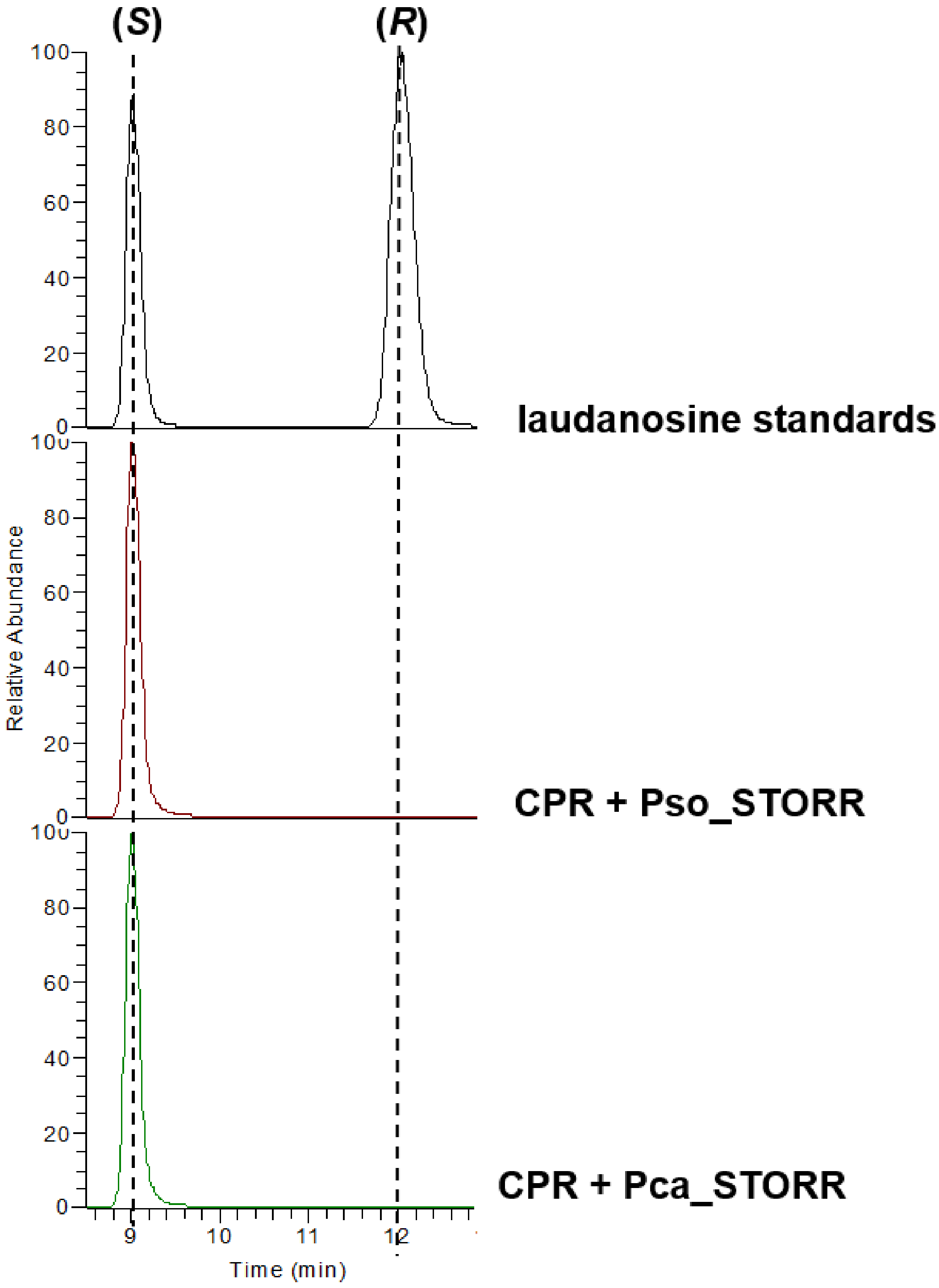

Supplementary Figure 4. Enzyme assay with (*S*)-laudanosine substrate

Substrate specificity of the microsomal extracts from *S. cerevisiae* expressing the STORR protein from *P. californicum* alongside *P. somniferum* (control) were assayed with (*S*)-laudanosine as a substrate. Relative abundance is used to show the laudanosine epimers present in the sample. Laudanosine standards (black), pESC-TRP+ CPR+ Pso *STORR* (red) and pESC-TRP+ CPR+ Pca *STORR* (green). Source data are provided as a Source Data file.

Supplementary Table 1. Plant material, origins and main metabolites identified from latex and capsule extractions.

Species	Source	Collected from	Accession/Voucher number	Major metabolite latex	Major metabolite capsule
Papaver setigerum	The Seed basket	Cranbrook BC, Canada	Poppy of Troy	Noscapine	Noscapine
Papaver rhoeas	Bonn Botanic Garden	Germany	DE-0-BONN-21621	-	m/z 384.14
Papaver dubium ssp. lecoqii	Bonn Botanic Garden	Germany	DE-0-BONN-23719	m/z 479.189	m/z 479.189
Papaver armeniacum	Ornamental plant germplasm center	Armenia	W6 23866 ARM 113	N methyl coclaurine	N methyl coclaurine
Papaver triniifolum	B and T	Paguignan, France	63504	m/z 296.129	m/z 296.129
Papaver bracteaum	Ornamental plant germplasm center	Iran	PI 374817 Voucher No. 793	Thebaine	Thebaine
Papaver orientale	Ornamental plant germplasm center	Iran	PI 253164	Thebaine	oripavine
Papaver atlanticum	Bonn Botanic Garden	unknown	xx-0-BONN-3315	Protopine	Rhoeadine
Papaver californicum	Rancho Santa Ana BG	California,	#21716	Glaucine	Glaucine
Papaver nudicaule	Ornamental plant germplasm center	Mongolia	W6 18131 E94216	m/z 332.187	m/z 332.187
Papaver pavoninum	GBIS_IPK_Gatersleben	Netherlands	PAP 546	m/z 2323.161	m/z 2323.161

Supplementary Table 2. RNAseq assemblies of *Papaver* species.

	Papaver pavoninum	*Papaver nudicaule*	*Papaver californicum*	*Papaver atlanticum*	*Papaver orientale*	*Papaver bracteatum*	*Papaver triniifolium*	*Papaver armeniacum*	*Papaver dubium*
**Tissue**	leaf	leaf	leaf	leaf	leaf	leaf	leaf	leaf	leaf
**Plant age (days)**	41	74	42	61	79	79	192	74	61
**Number of Illumina Paired- end Reads (x2)**	25671703	22080330	46031731	18482762	19743044	26709730	23365920	19974366	22411638
**Read length (bp)**	150	150	150	150	150	150	150	150	150
**Total trinity 'genes'**	109569	158235	118497	104318	174680	140779	113510	109308	139011
**Total trinity transcripts**	166330	238918	217245	165620	265609	220873	174915	172650	214565
**Average contig length (bp)**	592	537	986	630	509	587	628	626	542
**Contig prefix**	Ppa_	Pnu_	Pca_	Pat_	Por_	Pbr_	Ptr_	Par_	Pdu_

Supplementary Table 3. List of query sequences used to identify BIA biosynthetic pathway genes from transcriptomic datasets and draft genome assemblies.

Gene ID	Accession number	Gene description	BIA Pathway(s)
NCS	AAX56303.1	*S*-norcoclaurine synthase 1	(*S*)-Reticuline (precursor of berberine, sanguinarine, noscapine and morphinans)
6OMT	AAP45315.1	Norcoclaurine 6-*O*-methyltransferase	(*S*)-Reticuline (precursor of berberine, sanguinarine, noscapine and morphinans)
CNMT	AAP45316.1	Coclaurine *N*-methyltransferase	(*S*)-Reticuline (precursor of berberine, sanguinarine, noscapine and morphinans)
CYP80B1	AAF61400.1	(*S*)-*N*-methylcoclaurine 3'-hydroxylase	(*S*)-Reticuline (precursor of berberine, sanguinarine, noscapine and morphinans)
4OMT(4OMT1/4OMT2)	AAP45313.1/AAP45314.1	3'-hydroxy-*N*-methylcoclaurine 4'-*O*-methyltransferase 2	(*S*)-Reticuline (precursor of berberine, sanguinarine, noscapine and morphinans)
BBE	AAC61839.1	Berberine bridge enzyme	berberine, sanguinarine, noscapine
CFS	ADB89213.1	Cheilanthifoline synthase	sanguinarine
SPS	ADB89214.1	Stylopine synthase	sanguinarine
TNMT	AAY79177.1	(*S*)-tetrahydroprotoberberine-cis-N-methyltransferase	sanguinarine, noscapine
MSH	AGC92398.1	(*S*)-cis-N-methylstylopine 14-hydroxylase	sanguinarine
P6H	AGC92397.1	Protopine 6-hydroxylase	sanguinarine
CYP719A21	AFB74615.1	Canadine synthase	noscapine
PSMT1	AFB74611.1	*O*-methyltransferase 1	berberine, noscapine
CYP82Y1	AFB74617.1	Cytochrome P450, CYP82Y1	noscapine
CYP82X2	AFB74616.1	Cytochrome P450, CYP82X2	noscapine
PSAT1	AFB74620.1	Acetyltransferase 1	noscapine
CYP82X1	AFB74614.1	Cytochrome P450, CYP82X1	noscapine
PSMT2	AFB74612.1	*O*-methyltransferase 2	noscapine
PSMT3	AFB74613.1	*O*-methyltransferase 3	noscapine
PSCXE1	AFB74618.1	Carboxylesterase 1	noscapine
PSSDR1	AFB74619.1	Short-chain dehydrogenase/reductase	noscapine
STORR	AKN63431.1	(*S*)-to-(*R*)-reticuline P450-oxidoreductase	morphinans
SALSYN (CYP719B1)	ABR14720.1	Salutaridine synthase	morphinans
SALR	ABC47654.1	Salutaridine reductase	morphinans
SALAT	AAK73661.1	Salutaridinol 7-*O*-acetyltransferase	morphinans
THS2	AWQ63980.1	Thebaine synthase	morphinans

Supplementary Table 4. Summary of draft genome assemblies.

	Poppy Genomes in current manuscript					Published Genomes[14]		
	P. atlanticum	***P. californicum***	***P. nudicaule***	***P. bracteatum***	***P.armeniacum***	***P.somniferum***	***P. setigerum***	***P. rhoeas***
Sequencing Platform	10X Genomics			Oxford Nanopore Technology				
Genome assembly								
Estimated genome size by GenomeScope (GB)	1	1.3	1.4	2.2	NA			
Estimated heterozygosity by GenomeScope (%)	0.58	0.118	2.41	0.962	NA			
Assembled Genome size (Gb)	0.98	1.37	1.59	2.15	2.98	2.71	4.59	2.54
Average length of raw reads (bp)	150	150	150	13,395	22,216			
Contig N50 (Kb)	105.3	65	5.7	1.46	0.33			
Scaffold N50 (Mb)	3.42	4.38	0.0071	1.46	0.33	1.71	65.57	5.29
Largest scaffold (bp)	31,970,549	29,399,456	417,943	2,193,222	64,989			
Total number of Scaffolds	27,327	35,487	353,025	223,094	383,854			
LAI (LTR Assembly Index to assess genome assembly quality)	5.37	9.48	0	14.9	14.17			
Coding Gene Annotation								
Number of protein-coding genes	33,259	42,926	30,973	37,949	53,981	55,380	106,517	41,470
Supported by RNA-seq or homologs	95%	99%	99%	99%	99%	100%	100%	100%
Complete BUSCOs	96.30%	94.40%	71%	95.80%	95.10%	92.00%	95.90%	83.40%
Complete and single-copy BUSCOs	91.10%	46.30%	51.90%	88.60%	60.30%	30.50%	7.20%	71.10%
Complete and duplicated BUSCOs	5.20%	48.10%	19.10%	7.20%	34.80%	61.50%	88.70%	12.30%
Fragmented BUSCOs	1.90%	2.00%	14.60%	1.90%	2.50%	3.10%	1.10%	3.40%
Missing BUSCOs	1.80%	3.60%	16.40%	2.30%	2.40%	4.90%	3.00%	13.20%
WGD(s)	No WGD	one WGD		No WGD	One WGD	One WGD	Two WGDs	No WGD
