# Supplementary material for: A functionally conserved STORR gene fusion in Papaver species that diverged 16.8 million years ago
Source: Nat Commun. 2022 Jun 7;13:3150. doi: 10.1038/s41467-022-30856-w (PMC9174169; doi:10.1038/s41467-022-30856-w)
Supplement: Supplementary file 3 — Description of Additional Supplementary Files [file 41467_2022_30856_MOESM3_ESM.pdf]

Description of Additional Supplementary Files

File Name: Supplementary Data 1

Description: Metabolite analysis results for promorphinan and morphinan compounds. (Source data are provided as a Source Data file)

File Name: Supplementary Data 2

Description: Presence of selected orthologous BIA biosynthetic genes in transcriptomic datasets and genome assemblies of Papaver species. (Source data are provided as a Source Data file)

File Name: Supplementary Data 3

Description: List of eight conserved ortholog sets (COS) genes from the RNAseq assemblies of nine Papaver species as well as annotated genome assembles of six other Ranunculales species, A. coerulea, E. californica, M. cordata, P. rhoeas, P.setigerum and P. somniferum. (Source data are provided as a Source Data file)

File Name: Supplementary Data 4

Description: Percentage identity matrix between pairs of homologs of STORR, SALSYN, SALAT, SALR and THS in Papaver species. (Source data are provided as a Source Data file)

File Name: Supplementary Data 5

Description: Synteny analysis of STORR-containing contigs/scaffolds between opium poppy and other Papaver species. (Source data are provided as a Source Data file)

File Name: Supplementary Data 6

Description: Nucleotide sequences of yeast codon optimised full length STORR cDNAs from Papaver species.
