# Supplementary material for: A functionally conserved STORR gene fusion in Papaver species that diverged 16.8 million years ago
Source: Nat Commun. 2022 Jun 7;13:3150. doi: 10.1038/s41467-022-30856-w (PMC9174169; doi:10.1038/s41467-022-30856-w)
Supplement: Supplementary file 10 — Reporting Summary [file 41467_2022_30856_MOESM10_ESM.pdf]

nature portfolio

Corresponding author(s): Ian A. Graham

Last updated by author(s): 4th May 2022

Reporting Summary

Nature Portfolio wishes to improve the reproducibility of the work that we publish. This form provides structure for consistency and transparency in reporting. For further information on Nature Portfolio policies, see our Editorial Policies and the Editorial Policy Checklist.

Statistics

For all statistical analyses, confirm that the following items are present in the figure legend, table legend, main text, or Methods section.

n/a	Confirmed	
☒	☐	The exact sample size (*n*) for each experimental group/condition, given as a discrete number and unit of measurement
☒	☐	A statement on whether measurements were taken from distinct samples or whether the same sample was measured repeatedly
☒	☐	The statistical test(s) used AND whether they are one- or two-sided<br>*Only common tests should be described solely by name; describe more complex techniques in the Methods section.*
☒	☐	A description of all covariates tested
☒	☐	A description of any assumptions or corrections, such as tests of normality and adjustment for multiple comparisons
☐	☒	A full description of the statistical parameters including central tendency (e.g. means) or other basic estimates (e.g. regression coefficient) AND variation (e.g. standard deviation) or associated estimates of uncertainty (e.g. confidence intervals)
☒	☐	For null hypothesis testing, the test statistic (e.g. *F*, *t*, *r*) with confidence intervals, effect sizes, degrees of freedom and *P* value noted<br>*Give P values as exact values whenever suitable.*
☐	☒	For Bayesian analysis, information on the choice of priors and Markov chain Monte Carlo settings
☒	☐	For hierarchical and complex designs, identification of the appropriate level for tests and full reporting of outcomes
☒	☐	Estimates of effect sizes (e.g. Cohen's *d*, Pearson's *r*), indicating how they were calculated

Our web collection on statistics for biologists contains articles on many of the points above.

Software and code

Policy information about availability of computer code

Data collection	Genome and RNA sequencing were out-sourced and data were provided by service providers. No additional software was used for sequencing data collection.<br>Metabolite data was collected and analysed using the Thermo Xcalibur software (v 4.0.27.10)(Thermo Fisher Scientific Inc., Hemel Hempstead,UK) The morphinan alkaloids were identified by comparing exact mass and retention time values to those of authentic standards run under the same conditions. Data analysis for circular dichroism was performed with Jasco Spectra Manager v2 software.

Data analysis	Trinity (v2.2.0), RNA-Seq De novo Assembly; MUSCLE (v3.2), Multiple sequence alignment; Gblocks (v0.91), Selection of conserved blocks from multiple alignments for their use in phylogenetic analysis; BEAST2 (v2.5.1), Species phylogeny and estimation of divergence times; Tracer (v1.5), a tool for visualising and analysing the MCMC trace files generated through Bayesian phylogenetic inference; Figtree (v1.4.3), a graphical viewer of phylogenetic trees; EMBOSS Matcher (version 2.0u4), a pairwise sequence comparison tool; GenomeScope (v1.0), a tool for estimating genome size and heterozygosity level; Supernova (v2.1.1), a software package for de novo assembly from Chromium Linked-Reads that are made from a single whole-genome library from an individual DNA source; FLYE (version2.8-b1674), genome assembler for Oxford Nanopore sequencing datasets; Racon (v1.4.21), a polishing tool after the genome assembly; FREEBAYES (v1.3.5), a Bayesian genetic variant detector designed to find small polymorphisms; Purge_dups (v1.0), a tool for purging haplotigs and overlaps in an assembly based on read depth; Longranger (v2.2.2), a set of analysis pipelines that processes Chromium sequencing output to align reads and call and phase SNPs, indels, and structural variants; FGENESH (v2.1), a web-based gene annotation tool (http://www.softberry.com/berry.phtml?topic=fgenesh&group=programs&subgroup=gfind). RepeatModeler (v2.0.1, http://www.repeatmasker.org/RepeatModeler), repeat library construction. RepeatMasker (v4.1.1, http://www.repeatmasker.org), consensus TE sequences generation. LTR_retriever (v2.8), LTR_FINDER (v1.1) and LTR_harvest (v1.5.9), repetitive element identification. GeMoMa (v1.8, http://www.jstacs.de/index.php/GeMoMa), homology-based prediction. HISAT2 (v2.1.0) and StringTie2 (v2.1.5), mapping and aligning transcriptome assemblies. Transdecoder (v5.0.2, https://github.com/TransDecoder) identification of potential open reading frames. BRAKER2. EvidenceModeler (v1.1.1), gene annotation joining. InterProScan (v5.52-86), identification of protein-coding genes BUSCO version 4.1.4 and embryophyta_odb10.

For manuscripts utilizing custom algorithms or software that are central to the research but not yet described in published literature, software must be made available to editors and reviewers. We strongly encourage code deposition in a community repository (e.g. GitHub). See the Nature Portfolio guidelines for submitting code & software for further information.

Data

Policy information about availability of data

All manuscripts must include a data availability statement. This statement should provide the following information, where applicable:

- Accession codes, unique identifiers, or web links for publicly available datasets
- A description of any restrictions on data availability
- For clinical datasets or third party data, please ensure that the statement adheres to our policy

The transcript and genome assembly data generated in this study have been deposited in the NCBI databases under the BioProject PRJNA770669 (https://www.ncbi.nlm.nih.gov/bioproject/PRJNA770669). Raw RNA-seq and DNA reads are available under the accession numbers SRR16389878 (https://www.ncbi.nlm.nih.gov/sra/SRR16389878) - SRR16389886 (https://www.ncbi.nlm.nih.gov/sra/SRR16389886), SRR16690173 (https://www.ncbi.nlm.nih.gov/sra/SRR16690173) - SRR 16690174 (https://www.ncbi.nlm.nih.gov/sra/SRR16690174) and SRR16591802 (https://www.ncbi.nlm.nih.gov/sra/SRR16591802) - SRR16591806 (https://www.ncbi.nlm.nih.gov/sra/SRR16591806). Assembled RNA-seq data sets are available under the following accession numbers GJOO00000000 - GJOY00000000 and annotated genomes are under the following accession JAJJMA000000000 - JAJJMC000000000 and JAJJWW000000000 and JAJJWX000000000. Individual sequences of STORR and COR-L genes generated in this study have also been deposited at NCBI under the following accession numbers STORR = OK631703 - OK631704, Papaver nudicaule COR-L = OK631706 - OK631710, OK999969 - OK999970 and OL452058 .
Source data are provided with this paper and all data associated with this work are included in the manuscript and its Supplementary Information files.

Field-specific reporting

Please select the one below that is the best fit for your research. If you are not sure, read the appropriate sections before making your selection.

☒ Life sciences ☐ Behavioural & social sciences ☐ Ecological, evolutionary & environmental sciences

For a reference copy of the document with all sections, see nature.com/documents/nr-reporting-summary-flat.pdf

Life sciences study design

All studies must disclose on these points even when the disclosure is negative.

Sample size	Single individual plants were used for genome sequencing of each species as this is the standard approach. Leaf tissues for transcriptomic analysis were pooled from multiple individuals from the same species. Latex and capsule samples were collected for metabolite analysis from at least three plants of the same developmental stage for each species. A biological sample size of 3 was chosen to meet journal requirements. Three technical replications of the extracts were run on the UPLC.
Data exclusions	No data were excluded from the analysis.
Replication	No replication was needed for the genomic sequencing and RNA-Seq analysis for RNA-seq the data was used qualitatively to obtain gene sequences rather than quantitatively. Microsomal preparations from yeast cultures heterologously expressing STORR were carried out at least two times per construct except for Pbra_STORR which had been characterized previously. Subsequent measurement of STORR activities for each microsomal preparation was performed with three technical replicates, with all replicates being successful and producing the same results.
Randomization	Not applicable, no formal randomization was carried out. Randomization was not required for the genomic and transcriptomic analysis carried out.

Blinding	Not applicable, no blinding experiments were carried out for metabolite or genomic experiments as there would be no opportunity for operator actions to bias the outcomes.

Reporting for specific materials, systems and methods

We require information from authors about some types of materials, experimental systems and methods used in many studies. Here, indicate whether each material, system or method listed is relevant to your study. If you are not sure if a list item applies to your research, read the appropriate section before selecting a response.

Materials & experimental systems

n/a	Involved in the study
☒	☐ Antibodies
☒	☐ Eukaryotic cell lines
☒	☐ Palaeontology and archaeology
☒	☐ Animals and other organisms
☒	☐ Human research participants
☒	☐ Clinical data
☒	☐ Dual use research of concern

Methods

n/a	Involved in the study
☒	☐ ChIP-seq
☒	☐ Flow cytometry
☒	☐ MRI-based neuroimaging
